# Clinical and Radiographic Outcomes of Fibula-Preserving Supramalleolar Osteotomy Combined with Arthroscopic Modified Broström Operation in Varus Ankle Osteoarthritis

**DOI:** 10.3390/medicina62071221

**Published:** 2026-06-23

**Authors:** Ho-Sung Kim, Sung Hwan Kim, Young Koo Lee

**Affiliations:** 1 Department of Orthopaedic Surgery, Soonchunhyang University Bucheon Hospital, 170, Jomaru-ro, Bucheon 14584, Gyeonggi-do, Republic of Korea; nine4141@naver.com; 2 Department of Orthopaedic Surgery, Armed Forces Capital Hospital, Seongnam-si 13574, Gyeonggi-do, Republic of Korea; shk9528@naver.com

**Keywords:** ankle osteoarthritis, varus deformity, supramalleolar osteotomy, fibular osteotomy, arthroscopic modified Broström operation, joint-preserving surgery

## Abstract

*Background and Objectives*: Conventional supramalleolar osteotomy (SMO) often involves a concomitant fibular osteotomy (FO), which carries risks, such as nonunion and nerve injury. We evaluated the clinical and radiological outcomes of a fibula-preserving SMO (FP-SMO) combined with arthroscopic modified Broström operation (MBO) in patients with medial compartment varus ankle osteoarthritis and chronic lateral ankle instability. *Materials and Methods*: We retrospectively reviewed 22 patients who underwent medial opening wedge FP-SMO and arthroscopic MBO between 2014 and 2019. Clinical outcomes were assessed using the Visual Analog Scale (VAS), American Orthopaedic Foot and Ankle Society (AOFAS) Ankle-Hindfoot score, and Foot and Ankle Outcome Score (FAOS). Radiological evaluation included the anterior drawer test (ADT), talar tilt angle, tibiotalar surface (TTS) angle, medial distal tibial angle (MDTA), tibial lateral surface (TLS) angle, Takakura stage, and International Cartilage Regeneration and Joint Preservation Society (ICRS) grade assessed during second-look arthroscopy. *Results*: At a mean follow-up of 17.22 months, the mean VAS, AOFAS, and FAOS scores improved significantly (*p* < 0.001). Radiologically, the mean ADT decreased from 5.98 mm to 4.70 mm (*p* = 0.015), and the mean talar tilt angle decreased from 9.85° to 6.09° (*p* < 0.001). The mean TTS angle increased from 80.46° to 84.86° (*p* = 0.021), and the mean MDTA increased from 85.03° to 91.26° (*p* < 0.001). The TLS angle showed no significant change from 81.17° to 81.54° (*p* = 0.238). Takakura stage and ICRS grade improved or remained stable in all patients. No major complications, including nonunion, were observed. *Conclusions*: FP-SMO combined with arthroscopic MBO demonstrated favorable short-term clinical and radiological outcomes in selected patients with medial compartment varus ankle osteoarthritis and chronic lateral ankle instability. This combined approach may be a feasible joint-preserving option that addresses coronal malalignment and lateral ankle instability without requiring FO; however, longer-term comparative studies are needed to confirm its durability and clinical utility.

## 1. Introduction

Ankle osteoarthritis (OA) is a chronic condition affecting approximately 1% of the global population, with an estimated annual incidence of 30 cases per 100,000 individuals, representing only 2–4% of all OA cases [1]. Although its prevalence is lower than that of knee or hip OA, 75–80% of ankle OA cases are post-traumatic, often resulting from chronic ligamentous insufficiency [1,2,3,4]. Consequently, affected patients are typically younger and more active, most commonly between 18 and 44 years of age, and exhibit a tendency for rapid progression to end-stage OA within 10–20 years after the onset of degenerative changes. These characteristics emphasize the critical importance of establishing an appropriate treatment strategy [1,2,3,5]. This consideration is particularly relevant in physically active patients, for whom activity-related demands, including sports participation, further emphasize the need to preserve ankle range of motion, neuromuscular control, and functional stability [6,7].

Of the various patterns of ankle arthritis, medial compartment OA associated with varus deformity (varus OA) is the most prevalent [5,8,9,10]. Varus ankle OA arises from medial deviation of the ankle’s mechanical and anatomical axes, resulting in eccentric load distribution during weight bearing, asymmetric medial cartilage wear, and progressive joint incongruity [8,11,12]. Surgical interventions are broadly classified into joint-sacrificing procedures (JSP), including arthrodesis and total ankle arthroplasty, and joint-preserving procedures, which are aimed at realigning the joint while maintaining its anatomical integrity [1,2,8,13]. Although JSP can provide reliable pain relief, arthrodesis results in loss of joint motion and compensatory overload of adjacent joints, whereas total ankle arthroplasty raises concerns regarding mid- to long-term durability [1,14,15]. In young and active patients, joint-preserving procedures, such as supramalleolar osteotomy (SMO) are commonly used to mitigate the complications associated with JSP and prevent subsequent degenerative changes in adjacent joints [2,5,16,17,18,19].

The primary objective of conventional SMO is to redistribute load from the overloaded medial compartment to the relatively preserved lateral compartment through realignment of the distal tibia [11,20]. Traditionally, SMO has been performed in conjunction with arthroscopic procedures for intra-articular assessment and fibular osteotomy (FO) to facilitate deformity correction [5,21]. Even when the radiographic fibular length appears adequate, several studies have contended that concomitant FO is often required to overcome the limitations of isolated tibial correction and to achieve optimal joint congruence [14]. However, despite its routine use, the necessity of FO remains debated, as it prolongs operative time and introduces additional risks, including nonunion and peroneal nerve injury [11,22]. Therefore, when adequate correction can be achieved without fibular osteotomy, fibular preservation may offer a less invasive, tissue-sparing alternative while maintaining the lateral stabilizing structures. Recent finite element analysis has suggested that fibula-preserving SMO (FP-SMO) can achieve adequate redistribution of joint contact pressure [11]. In addition, clinical cohort studies have reported favorable outcomes after distal tibial osteotomy without fibular osteotomy in selected patients [23]. Moreover, preservation of the intact lateral fibula may provide a stable lateral hinge, enhancing mechanical stability during opening of the osteotomy gap [24,25].

Furthermore, given the underlying pathophysiology of varus ankle OA, comprehensive management should address osseous alignment and intra-articular pathology and periarticular soft-tissue structures. Chronic ligamentous instability accounts for approximately 16% of cases of varus ankle OA [15,16]. Such instability increases peak joint contact stress and accelerates cartilage degeneration, even when present as microinstability [2,15,16]. Chronic lateral ligament insufficiency, which is frequently observed in patients with varus OA, induces talar tilt and exacerbates medial load concentration [5,14]. Therefore, in cases accompanied by lateral instability, restoration of ligamentous tension through the modified Broström operation (MBO) may contribute to maintenance of the corrected alignment and improvement of ankle joint mechanical stability [14]. Restoration of lateral ankle stability through a MBO combined with arthroscopic debridement results in meaningful clinical and radiologic improvement in osteoarthritic symptoms [16]. Transition from conventional open techniques to an arthroscopic MBO may reduce soft-tissue morbidity while enabling the simultaneous management of intra-articular pathology [26].

We evaluated the clinical and radiologic outcomes of FP-SMO combined with arthroscopic debridement and MBO in selected patients with medial compartment varus ankle OA and chronic lateral ankle instability. This approach was intended to address distal tibial coronal malalignment, intra-articular pathology, and lateral ligamentous instability while avoiding routine FO. We sought to determine whether FP-SMO combined with arthroscopic MBO represents a feasible joint-preserving strategy in this patient population.

## 2. Materials and Methods

### 2.1. Study Population and Design

The Institutional Review Board of our institution approved the study protocol (approval no. 2026-03-007). The study was performed in accordance with the Declaration of Helsinki. We conducted a retrospective review of patients who underwent FP-SMO combined with arthroscopic MBO for medial compartment varus ankle OA between January 2014 and December 2019.

**Figure 1:**
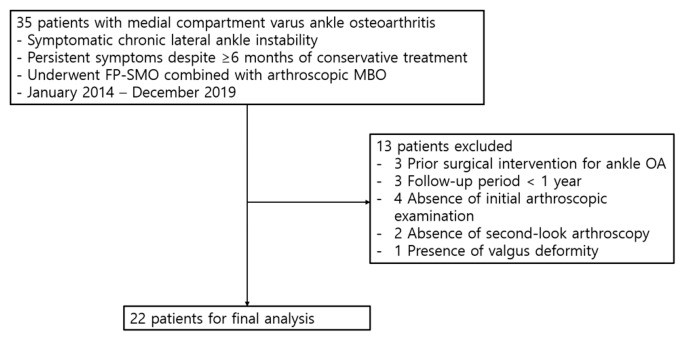
Flowchart detailing the patient selection process for the study.

In total, 35 patients were initially identified. Patients were eligible if they had medial compartment varus ankle OA with symptomatic chronic lateral ankle instability and had persistent symptoms despite at least 6 months of conservative treatment. Chronic lateral ankle instability was defined by symptomatic giving way or recurrent instability, together with supportive findings on physical examination, such as a positive anterior drawer test, and/or increased anterior talar translation or talar tilt on stress radiographs. To ensure a homogeneous study population and facilitate objective comparison of intra-articular status based on second-look arthroscopic findings, patients were excluded if they had undergone prior surgical intervention for ankle OA, had a follow-up duration of <1 year, lacked an initial arthroscopic evaluation at the time of the index surgery, lacked second-look arthroscopic assessment of cartilage during plate removal, or had other deformities, including valgus alignment. After applying these criteria, 22 patients were included in this single-arm observational study. Written informed consent was obtained from all participants or their legal guardians before study initiation (Figure 1).

### 2.2. Surgical Management

A medial opening-wedge FP-SMO was performed initially. A 5-cm longitudinal skin incision was made along the medial malleolus to expose the distal tibial metaphysis. Under fluoroscopic guidance, a 1.8-mm Kirschner wire was inserted from the medial aspect, approximately 4–5 cm proximal to the joint line, and directed toward the proximal one-third of the intra-syndesmotic region, which is considered the “safe zone” for the lateral hinge, to minimize the risk of lateral cortical fracture [25]. The osteotomy was initiated using an oscillating saw and was completed carefully with a thin osteotome, ensuring preservation of the lateral cortex to maintain a stable hinge. In our study, FO was avoided to preserve lateral stabilizing structures and avoid additional FO-related morbidity, such as fibular nonunion or hardware irritation [24].

**Figure 2:**
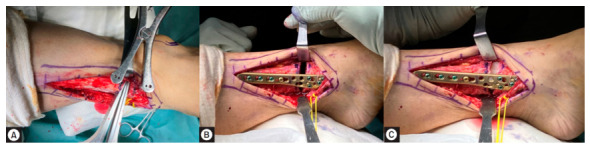
Intraoperative gross photographs demonstrating the sequence of the medial opening wedge fibula-preserving supramalleolar osteotomy (FP-SMO). (**A**) Initial opening of the osteotomy site. (**B**) Maintenance of the targeted osteotomy gap. (**C**) Filling of the osteotomy gap with allogeneic bone graft.

**Figure 3:**
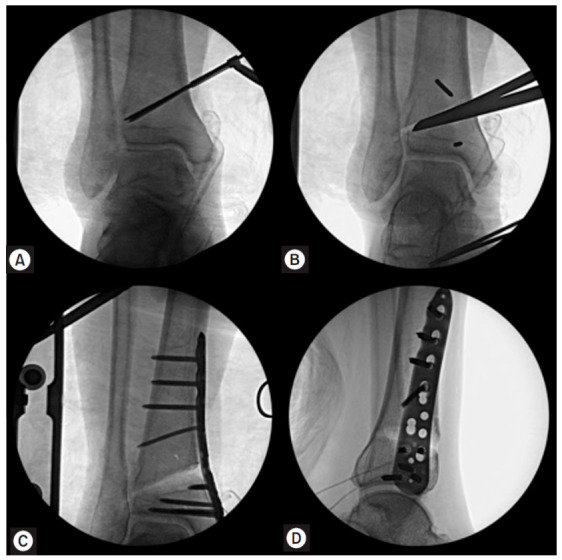
Intraoperative fluoroscopic images demonstrating the sequence of the medial opening wedge fibula-preserving supramalleolar osteotomy (FP-SMO). (**A**) Guide pin insertion and targeting for the osteotomy. (**B**) Gradual opening of the medial osteotomy site using a spreader, demonstrating the preservation of the intact lateral hinge and the fibula. (**C**) Final anteroposterior and (**D**) lateral views showing stable fixation with a locking compression plate and bone grafting.

The osteotomy site was opened using a lamina spreader until the desired correction was achieved, with the goal of realigning the tibial anatomical axis with the talar dome (Figure 2). Preservation of the lateral hinge during FP-SMO allowed for controlled correction of the varus deformity while maintaining mechanical stability. After coronal and sagittal alignment was confirmed under anteroposterior and lateral fluoroscopy, the wedge gap was measured. The osteotomy gap was then filled with allogeneic bone graft. Definitive internal fixation was performed using a 3.5-mm locking compression plate (LCP; DePuy Synthes, West Chester, PA, USA) to provide rigid stabilization (Figure 3).

After completion of FP-SMO and confirmation of stable fixation, diagnostic ankle arthroscopy was performed through standard anteromedial and anterolateral portals. Intra-articular lesions, including synovitis and loose bodies, were addressed. In patients with cartilage defects, debridement or microfracture was performed as indicated.

**Figure 4:**
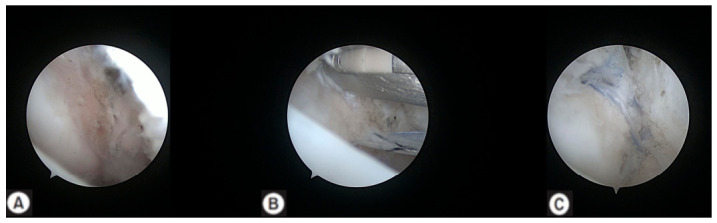
Intraoperative arthroscopic views demonstrating the sequence of the arthroscopic modified Broström operation (MBO). (**A**) Debridement and preparation of the fibular footprint for anatomic ligament repair. (**B**) Insertion of a suture anchor into the prepared fibular footprint. (**C**) Capsuloligamentous suture fixation with appropriate tensioning to restore lateral ankle stability.

Subsequently, lateral ligament repair was performed using arthroscopic MBO to restore mechanical alignment and soft-tissue stability. To prepare the footprint for anatomic repair, the accessory fibers of the distal tibiofibular ligament were resected at their fibular insertion. The synovial tissue and periosteum immediately distal to the anterior tibiofibular ligament were meticulously debrided using a motorized burr to expose a bleeding bony surface, facilitating biological healing. A drill hole was created perpendicular to the anterior surface of the fibula, and an absorbable Bio-SutureTak anchor (Arthrex, Naples, FL, USA), loaded with FiberWire and TigerWire, was inserted through the anterolateral portal. For precise suture management, an accessory anteroinferior portal was established in the sinus tarsi region, and a far-lateral portal was created over the anterior fibula. A penetrator was used to pass one limb of the suture intra-articularly from the anterolateral portal to the accessory anteroinferior portal, while the other limb was retrieved through the far-lateral portal. Subsequently, a suture retriever was advanced from the far-lateral portal to the accessory anteroinferior portal, to shuttle the sutures subcutaneously, incorporating the lateral capsuloligamentous structures. Final knot tying was performed with the foot maintained in eversion and dorsiflexion to achieve appropriate tensioning and restore stability of the lateral ankle complex (Figure 4) [26].

### 2.3. Postoperative Rehabilitation Protocol

Immediately after surgery, a short leg splint was applied, and patients were permitted 50% partial weight-bearing. At 2 weeks postoperatively, after confirmation of uneventful wound healing, sutures were removed and the splint was replaced with a short leg cast. To accommodate the reduction in postoperative swelling, the cast was changed at 4 weeks. The short leg cast was maintained until 6 weeks postoperatively.

At 6 weeks, the cast was removed and patients were transitioned to a controlled ankle motion (CAM) walker boot. Concurrently, range of motion, proprioceptive, and peroneal muscle-strengthening exercises were initiated. During this period (6–8 weeks), the CAM boot was worn continuously and removed only temporarily for home-based ROM exercises.

At 8 weeks postoperatively, full weight-bearing was permitted with the use of a CAM walker boot, and patients were instructed to initiate heel-raising and squatting exercises. Thereafter, the CAM boot was reserved for outdoor ambulation and discontinued during indoor activities.

At approximately 3 months postoperatively, jumping exercises were introduced. The CAM boot was discontinued once the patient was able to perform three consecutive jumps without difficulty. Progression through each phase of the rehabilitation protocol was permitted only after confirmation of the absence of wound complications and abnormal radiographic findings. The rehabilitation protocol was designed to protect the osteotomy site and repaired lateral ligament complex while progressively restoring ankle range of motion, proprioception, and dynamic stability. Proprioceptive training and peroneal strengthening were emphasized because residual deficits in proprioception, peroneal strength, and postural control may persist after lateral ligament repair, and targeted strengthening exercises may improve joint position sense in patients with chronic ankle instability [27,28].

### 2.4. Clinical and Radiological Outcome Assessment

Clinical outcomes were assessed preoperatively and at the final follow-up using the Visual Analog Scale (VAS) for pain, the American Orthopaedic Foot and Ankle Society (AOFAS) Ankle-Hindfoot Scale, and the Foot and Ankle Outcome Score (FAOS).

Radiological parameters were evaluated using weight-bearing and stress radiographs. The severity of ankle OA was classified according to the Takakura staging system. Joint alignment was quantified by measuring the tibiotalar surface (TTS) angle on weight-bearing radiographs, and distal tibial coronal alignment was assessed using the medial distal tibial angle (MDTA) on weight-bearing anteroposterior radiographs. Sagittal alignment was evaluated using the tibial lateral surface (TLS) angle on weight-bearing lateral radiographs. Lateral ankle instability was assessed using the anterior drawer test (ADT, mm) on stress radiographs, and coronal-plane instability was further evaluated using the talar tilt angle on varus stress radiographs. These radiological parameters were measured preoperatively and at 6 months, 12 months, and the final follow-up. After radiographic union of the osteotomy site was confirmed, plate removal with second-look arthroscopy was performed approximately 1 year postoperatively. Intra-articular cartilage status was directly visualized during both the index procedure and second-look arthroscopy and was graded according to the International Cartilage Regeneration and Joint Preservation Society (ICRS) classification system. In this study, an improvement in cartilage status was defined as a decrease of at least one grade in the ICRS classification between the index surgery and second-look arthroscopy. To ensure objectivity and minimize observer bias, radiographic osteoarthritic stage and arthroscopic cartilage status were independently assessed by two orthopaedic surgeons (Evaluator 1 and Evaluator 2; a foot and ankle specialist and a third-year resident), who were blinded to the patients’ clinical information and outcomes. Radiographic osteoarthritic stage was evaluated using serial radiographs, whereas ICRS grade was assessed using intraoperative arthroscopic images from the index procedure and second-look arthroscopy stored in the PACS. The results from both evaluators were presented separately to demonstrate the consistency of the findings.

### 2.5. Statistical Analysis

Statistical analyses were performed using Rex software (version 3.0.3; RexSoft, Seoul, Republic of Korea). Continuous variables were expressed as the mean ± standard deviation. After confirming the normality of the data distribution using the Shapiro-Wilk test, preoperative and postoperative clinical and radiological parameters were compared using the paired *t*-test. Inter-observer reliability for the ICRS grading was assessed by calculating the intraclass correlation coefficient (ICC) with 95% confidence intervals. *p*-values < 0.05 were considered statistically significant.

## 3. Results

### 3.1. Patient Demographics

In total, 35 patients were initially assessed for eligibility. After application of the exclusion criteria, 22 patients (22 ankles) who underwent FP-SMO combined with arthroscopic MBO were included in the final analysis. The cohort comprised 5 male and 17 female patients, with a mean age of 60.9 ± 9.8 years at the time of surgery and a mean BMI of 25.77 ± 3.82 kg/m^2^. The mean follow-up duration was 17.22 (11.7–41.8) months. Detailed patient characteristics are summarized in Table 1.

### 3.2. Clinical Outcomes

**Figure 5:**
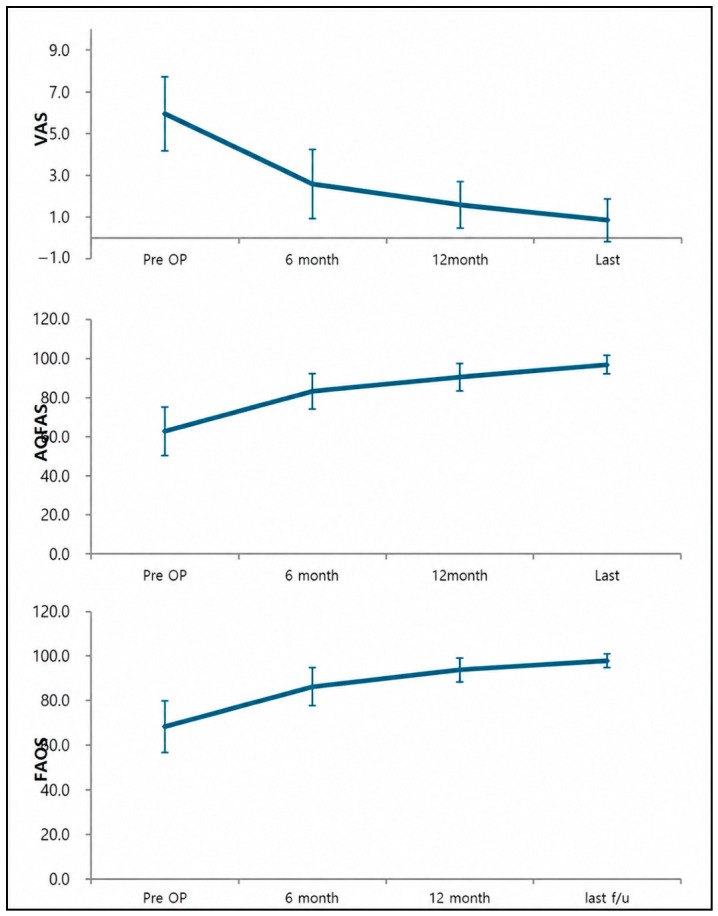
Serial changes in clinical outcomes. The graphs demonstrate significant postoperative improvements in the Visual Analog Scale (VAS) for pain, the American Orthopaedic Foot and Ankle Society (AOFAS) Ankle-Hindfoot Scale, and the Foot and Ankle Outcome Score (FAOS) at 6 months, 12 months, and the last follow-up compared to the preoperative baseline.

All subjective clinical scores improved significantly postoperatively. The mean VAS score decreased from 5.95 ± 1.76 preoperatively to 0.86 ± 1.01 at the final follow-up (*p* < 0.001). The mean AOFAS Ankle-Hindfoot score increased from 62.77 ± 12.29 preoperatively to 96.76 ± 4.70 at the final follow-up (*p* < 0.001). Similarly, the mean FAOS increased from 68.55 ± 11.46 preoperatively to 98.00 ± 3.02 at the final follow-up (*p* < 0.001). Significant improvements in VAS, AOFAS, and FAOS were observed at 6 and 12 months postoperatively compared to preoperative values (*p* < 0.001; Table 2; Figure 5).

### 3.3. Radiological Outcomes

**Figure 6:**
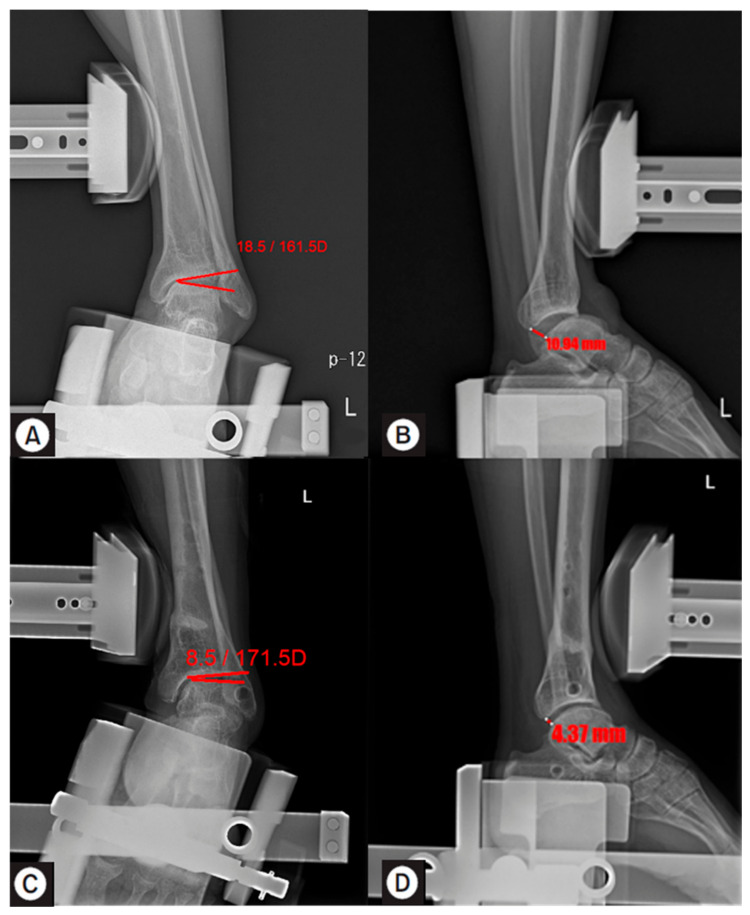
Representative preoperative and postoperative stress radiographs. (**A**) Preoperative varus stress radiograph showing lateral ligamentous insufficiency with increased talar tilt. (**B**) Preoperative anterior drawer test (ADT) radiograph demonstrating anterior talar translation. (**C**) Postoperative varus stress radiograph at the last follow-up demonstrating reduced talar tilt. (**D**) Postoperative ADT radiograph showing decreased anterior talar translation following the arthroscopic modified Broström operation (MBO). Red lines indicate the measured talar tilt angle and anterior talar translation; ‘L’ denotes the left-side radiographic marker.

**Figure 7:**
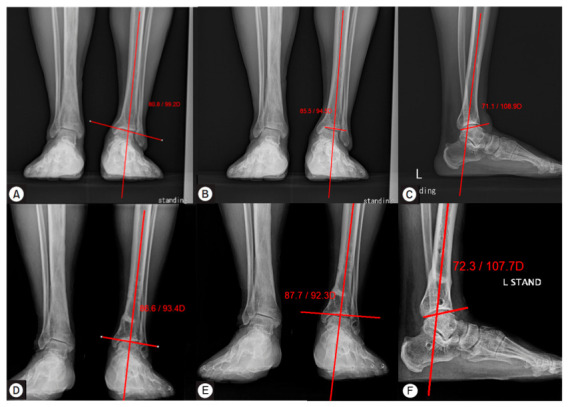
Representative preoperative and postoperative weight-bearing radiographs demonstrating coronal and sagittal alignment measurements. (**A**) Preoperative anteroposterior radiograph with the measured tibiotalar surface (TTS) angle, demonstrating varus ankle osteoarthritis. (**B**) Preoperative anteroposterior radiograph with the measured medial distal tibial angle (MDTA), demonstrating distal tibial coronal alignment. (**C**) Preoperative lateral radiograph with the measured tibial lateral surface (TLS) angle, demonstrating sagittal alignment. (**D**) Postoperative anteroposterior radiograph at the last follow-up showing correction of the TTS angle, successful correction of the varus deformity, and preservation of the intact lateral fibula following medial opening wedge fibula-preserving supramalleolar osteotomy (FP-SMO). (**E**) Postoperative anteroposterior radiograph at the last follow-up showing correction of the MDTA. (**F**) Postoperative lateral radiograph at the last follow-up showing maintained TLS angle and sagittal alignment. Red lines indicate the radiographic measurement lines for TTS, MDTA, and TLS angles.

**Figure 8:**
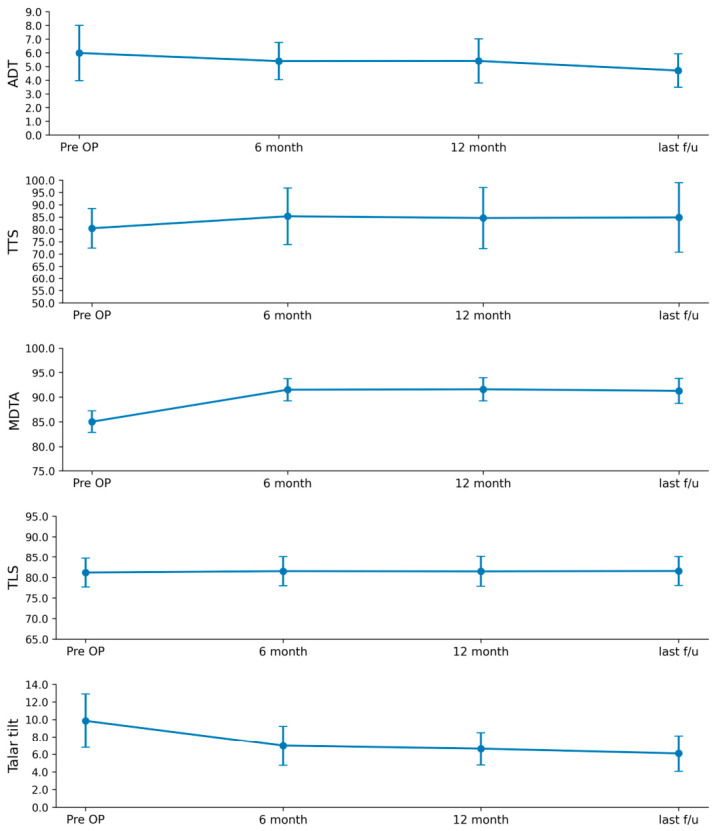
Serial changes in radiological outcomes. The graphs illustrate sequential postoperative changes in the anterior drawer test (ADT), tibiotalar surface (TTS) angle, medial distal tibial angle (MDTA), tibial lateral surface (TLS) angle, and talar tilt angle.

The mean ADT value decreased significantly from 5.98 ± 2.02 mm preoperatively to 4.70 ± 1.23 mm at the final follow-up (*p* = 0.015) (Figure 6). The mean talar tilt angle also decreased significantly from 9.85 ± 3.06° preoperatively to 6.09 ± 2.02° at the final follow-up (*p* < 0.001), indicating improvement in lateral ankle laxity on varus stress radiographs. The mean TTS angle increased significantly from 80.46 ± 8.05° preoperatively to 84.86 ± 14.07° at the final follow-up (*p* = 0.021). The mean MDTA increased significantly from 85.03 ± 2.19° preoperatively to 91.26 ± 2.53° at the final follow-up (*p* < 0.001), whereas the mean TLS angle showed no significant change from 81.17 ± 3.54° to 81.54 ± 3.51° (*p* = 0.238) (Figure 7). Serial changes in all radiological parameters are summarized in Table 2 and Figure 8.

### 3.4. Joint and Cartilage Status

At the final follow-up, both independent evaluators observed preservation or improvement of joint and cartilage status. According to Evaluator 1, the Takakura stage improved in 14 patients and was maintained in 8, while Evaluator 2 observed improvement in 11 patients and maintenance in 11. No cases of osteoarthritis progression were observed by either evaluator. Postoperatively, the ICRS grade improved or was maintained in all patients, with Evaluator 1 reporting improvement in 100% of cases and Evaluator 2 in 95.5%. Regarding the magnitude of cartilage improvement, Evaluator 1 observed 1-grade, 2-grade, and 3-grade improvements in 12, 9, and 1 patient, respectively, whereas Evaluator 2 observed 1-grade and 2-grade improvements in 15 and 6 patients, respectively, and maintenance in 1 patient. The overall inter-observer reliability for all evaluations was excellent (ICC = 0.858 for Takakura stage; ICC = 0.925 for ICRS grade). The detailed distributions of the grades from both evaluators are summarized in Table 3 and Table 4.

### 3.5. Complications

No major postoperative complications, including deep infection, neurovascular injury, or nonunion at the osteotomy site, were observed during the follow-up period. No delayed union, lateral hinge fracture, hardware irritation requiring unplanned intervention, postoperative nerve symptoms, wound complications, or reoperations were recorded. Serial radiographic assessment showed no meaningful loss of correction from 6 months to the final follow-up, as reflected by maintenance of the TTS angle, MDTA, and TLS angle.

## 4. Discussion

The principal finding of our study is that medial opening wedge FP-SMO combined with arthroscopic evaluation, intra-articular management, and arthroscopic MBO demonstrated favorable short-term clinical and radiological outcomes in selected patients with medial compartment varus ankle OA and chronic lateral ankle instability. All subjective clinical scores, including the VAS, AOFAS Ankle-Hindfoot score, and FAOS, improved significantly postoperatively. Radiologically, the TTS angle and MDTA improved significantly, the ADT value and talar tilt angle decreased significantly, and the TLS angle remained stable throughout follow-up. These findings suggest correction of coronal malalignment and lateral ankle laxity without evident unintended sagittal plane alteration. Joint and cartilage status, assessed using the Takakura staging system and ICRS grading system, also showed preservation or improvement, with no progression of osteoarthritis during the follow-up period.

Traditionally, a concomitant FO is often performed during conventional SMO to compensate for the limitations of isolated tibial correction and achieve optimal joint congruence [14]. However, its necessity remains controversial, as FO can increase operative time and is associated with additional risks, including nonunion and peroneal nerve injury [11]. Biomechanical and finite element analyses have suggested that FP-SMO may adequately redistribute ankle joint contact pressure without routine FO [11], while clinical cohort studies have reported favorable outcomes after distal tibial osteotomy without fibular osteotomy in selected patients [23]. In our study, the mean TTS angle improved significantly from 80.46° to 84.86°, and the mean MDTA improved from 85.03° to 91.26° without performing FO. Although this single-arm study does not allow direct comparison with conventional SMO with FO, these findings support the feasibility of FP-SMO as a tissue-sparing joint-preserving option in selected patients when adequate correction can be achieved without fibular osteotomy. In addition, no FO-related complications, such as fibular nonunion or peroneal nerve injury, were observed in this cohort.

To contextualize the present findings, selected previous studies addressing SMO, fibular osteotomy, and radiological assessment in varus ankle OA are summarized in Table 5.

Regarding osteotomy gap management, the absence of a structural graft in an opening wedge procedure raises concerns about gap subsidence, loss of correction, and delayed union. However, in our technique, the intentionally preserved lateral fibula acted as a stable hinge, providing intrinsic support [24,25]. In addition, internal fixation with a 3.5-mm LCP conferred rigid mechanical stabilization. This combination of intrinsic and extrinsic stability enabled the use of allogeneic bone graft without structural bone grafting and was associated with uneventful bone healing in this cohort. It also provided sufficient stability to allow early partial weight-bearing in the postoperative rehabilitation protocol. This early mobilization strategy may help reduce postoperative stiffness without compromising osteotomy integrity, as no delayed union, nonunion, lateral hinge fracture, or meaningful loss of correction from 6 months to the final follow-up was observed in the present cohort. The maintenance of TTS angle, MDTA, and TLS angle during follow-up further supports the short-term radiographic stability of this construct. Nevertheless, these findings reflect short-term stability in a selected cohort and do not establish long-term durability.

Furthermore, comprehensive management of varus ankle OA must address concomitant intra-articular pathology and lateral ligamentous instability. Chronic ligamentous instability has been reported in approximately 16% of varus ankle OA cases, increasing peak joint contact stress and accelerating cartilage degeneration, even in cases of microinstability [2,15,16]. In our study, the addition of arthroscopic MBO was associated with improved lateral ankle stability, as reflected by significant reductions in the mean ADT value from 5.98 to 4.70 mm and the mean talar tilt angle from 9.85° to 6.09°. Correcting this instability and associated talar tilt is essential for maintaining the realigned ankle and enhancing overall mechanical stability [14]. Simultaneous arthroscopic evaluation and targeted management of intra-articular lesions enabled direct treatment of concomitant intra-articular lesions and may have contributed to symptomatic improvement [1,29,30]. This integrated approach is corroborated by second-look arthroscopy, which demonstrated improvement or stability in ICRS grades in all 22 patients, while the Takakura stage was improved or maintained, with no progression of osteoarthritis. Using an arthroscopic technique further minimized soft tissue morbidity while achieving satisfactory mechanical stabilization and intra-articular management [26]. However, these arthroscopic findings should be interpreted as short-term improvement or stabilization of cartilage status rather than definitive evidence of durable cartilage regeneration.

Our study has several limitations. First, it is a retrospective observational study with a relatively small cohort of 22 patients and no direct control group, representing Level IV evidence. Therefore, our findings should be interpreted with caution. In particular, because no control group undergoing conventional SMO with FO was included, this study cannot determine whether FP-SMO provides superior radiographic correction, fewer complications, or better clinical outcomes compared with conventional SMO with FO.Second, an a priori power analysis was not performed because of the retrospective fixed-cohort design. Therefore, the study may be underpowered to detect small differences in radiological parameters or uncommon complications. Third, the mean follow-up of 17.22 months is relatively short. Therefore, we cannot definitively conclude the long-term durability of cartilage status improvement or the prevention of OA progression. Furthermore, short-term radiographic stability does not necessarily equate to long-term joint preservation. Fourth, time to union was not quantitatively analyzed because of the retrospective nature of the study, although no delayed union or nonunion was observed. Further large-scale, prospective comparative studies with extended follow-up are needed to confirm the long-term clinical efficacy, radiographic durability, and appropriate indications of this joint-preserving approach.

## 5. Conclusions

In our study cohort, medial opening wedge FP-SMO combined with arthroscopic MBO demonstrated favorable short-term clinical and radiological outcomes as a joint-preserving strategy for selected patients with medial compartment varus ankle OA and chronic lateral ankle instability. By addressing bony malalignment and lateral ligamentous instability without performing a FO, this approach may represent a feasible tissue-sparing option that maintains short-term radiographic correction and ankle stability. However, further long-term, prospective comparative studies are necessary to confirm the durability, clinical utility, and appropriate indications of this joint-preserving strategy.

## Figures and Tables

**Table 1:** Patient demographics.

Variable	Value (*n* = 22)
Age (yr)	60.91 ± 9.8
BMI (kg/m^2^)	25.77 ± 3.82
Sex	
Male	5 (22.73%)
Female	17 (77.27%)
Involved side	
Right	9 (40.91%)
Left	13 (59.09%)

Data are presented as mean ± standard deviation or number (percentage).

**Table 2:** Serial changes in clinical and radiological outcomes.

Variables	Preoperative	6 Months Follow-Up	12 Months Follow-Up	Last Follow-Up	*p*-Value
Clinical Outcomes					
VAS	5.95 ± 1.76	2.59 ± 1.65	1.59 ± 1.10	0.86 ± 1.01	<0.001
AOFAS	62.77 ± 12.29	83.18 ± 8.82	90.36 ± 6.81	96.76 ± 4.70	<0.001
FAOS	68.55 ± 11.46	86.50 ± 8.52	93.91 ± 5.26	98.00 ± 3.02	<0.001
Radiological outcomes					
TTS angle (°)	80.46 ± 8.05	85.33 ± 11.50	84.61 ± 12.43	84.86 ± 14.07	0.021
MDTA (°)	85.03 ± 2.19	91.49 ± 2.23	91.58 ± 2.36	91.26 ± 2.53	<0.001
TLS angle (°)	81.17 ± 3.54	81.50 ± 3.55	81.45 ± 3.64	81.54 ± 3.51	0.238
ADT (mm)	5.98 ± 2.02	5.39 ± 1.36	5.40 ± 1.61	4.70 ± 1.23	0.015
Talar tilt (°)	9.85 ± 3.06	6.98 ± 2.24	6.63 ± 1.86	6.09 ± 2.02	<0.001

Data are presented as mean ± standard deviation. *p*-values indicate the significance of differences between preoperative and last follow-up values. ADT: anterior drawer test, AOFAS: American Orthopaedic Foot and Ankle Society, FAOS: Foot and Ankle Outcome Score, MDTA: medial distal tibial angle, TLS: tibial lateral surface angle, TTS: tibiotalar surface, VAS: Visual Analog Scale.

**Table 3:** Serial Changes in Joint and Cartilage Status (Evaluator 1).

Variables	Preoperative	Last Follow-Up
Takakura stage		
Stage 1	0	7
Stage 2	12	12
Stage 3a	10	3
Overall change		
Improved	-	14 (63.6%)
Maintained	-	8 (36.4%)
Aggravated	-	0 (0%)
ICRS grade		
Grade 1	0	4
Grade 2	2	10
Grade 3	3	8
Grade 4	17	0
Magnitude of ICRS grade change		
Improved by 1 grade	-	12 (54.5%)
Improved by 2 grades	-	9 (40.9%)
Improved by 3 grades	-	1 (4.5%)
Maintained	-	0 (0%)
Aggravated	-	0 (0%)

Values are presented as number of ankles (percentage). ICRS: International Cartilage Regeneration & Joint Preservation Society.

**Table 4:** Serial Changes in Joint and Cartilage Status (Evaluator 2).

Variables	Preoperative	Last Follow-Up
Takakura stage		
Stage 1	0	4
Stage 2	12	15
Stage 3a	10	3
Overall change		
Improved	-	11 (50.0%)
Maintained	-	11 (50.0%)
Aggravated	-	0 (0%)
ICRS grade		
Grade 1	0	1
Grade 2	2	10
Grade 3	3	11
Grade 4	17	0
Magnitude of ICRS grade change		
Improved by 1 grade	-	15 (68.2%)
Improved by 2 grades	-	6 (27.3%)
Improved by 3 grades	-	0 (0%)
Maintained	-	1 (4.5%)
Aggravated	-	0 (0%)

Values are presented as number of ankles (percentage). ICRS: International Cartilage Regeneration & Joint Preservation Society.

**Table 5:** Summary of Selected Studies Relevant to Supramalleolar Osteotomy and Radiological Assessment.

Study/Design	Sample/Follow-Up	Procedure/Adjunctive Procedure	Radiological Focus	Key Relevance
Butler et al. [3]/systematic review	1160 patients/1182 ankles; weighted mean 50.4 months	Various SMO techniques; FO and adjuvant procedures varied	Clinical and radiographic outcomes; complications; failures	Overall evidence for favorable SMO outcomes and low complication rates.
Lee et al. [11]/finite element analysis	4 three-dimensional ankle models; follow-up not applicable	Simulated SMO alone versus SMO with FO; no ligament procedure	Peak medial tibiotalar contact pressure	Supports the biomechanical rationale for SMO without routine FO.
Ahn et al. [23]/retrospective cohort	18 patients/18 ankles; mean 34 months	Distal tibial osteotomy without FO; no planned ligament procedure	Talar translation; talar tilt; MDTA; ADTA	Supports the feasibility of tibial osteotomy without FO in selected patients.
Suh et al. [22]/retrospective cohort	47 patients/48 ankles; mean 4.0 years	Oblique SMO without FO; no planned ligament procedure	TAS; talar tilt; TTS; TLS; talofibular gaps; Takakura stage	Supports SMO without FO and emphasizes mortise congruence assessment.
Krähenbühl et al. [18]/retrospective cohort	44 patients/44 ankles; mean 40 months	Extra-articular medial opening wedge SMO; FO and ligament reconstruction performed selectively	TAS; TTS; talar tilt; TLS; sagittal talar translation; hindfoot alignment	Supports multidimensional radiographic assessment and selective adjunctive procedures.
Gong et al. [12]/retrospective radiographic study	70 patients; mean 28.8 months in main SMO group	SMO for varus ankle OA; FO performed in all patients	TAS; talar tilt; TTS; TTD; TTDP	Shows the limitation of relying on a single tibial surface angle.
Present study/retrospective single-arm study	22 patients/22 ankles; mean 17.22 months	Medial opening wedge FP-SMO combined with arthroscopic MBO	TTS; MDTA; TLS; ADT; talar tilt; Takakura stage; ICRS grade	Evaluates combined FP-SMO and arthroscopic MBO using expanded radiographic and arthroscopic outcomes.

Abbreviations: ADT, anterior drawer test; ADTA, anterior distal tibial angle; FO, fibular osteotomy; FP-SMO, fibula-preserving supramalleolar osteotomy; ICRS, International Cartilage Regeneration and Joint Preservation Society; MBO, modified Broström operation; MDTA, medial distal tibial angle; OA, osteoarthritis; SMO, supramalleolar osteotomy; TAS, tibial anterior surface angle; TLS, tibial lateral surface angle; TTD, distance from the center of the talus to the tibial axis; TTDP, percentage of talar center displacement; TTS, tibiotalar surface.

## Data Availability

The data presented in this study are available on request from the corresponding author. The data are not publicly available due to privacy and ethical restrictions.

## Abbreviations

The following abbreviations are used in this manuscript:

ADT	Anterior Drawer Test
AOFAS	American Orthopaedic Foot and Ankle Society
CAM	Controlled Ankle Motion
FAOS	Foot and Ankle Outcome Score
FO	Fibular Osteotomy
FP-SMO	Fibula-Preserving Supramalleolar Osteotomy
ICC	Intraclass Correlation Coefficient
ICRS	International Cartilage Regeneration and Joint Preservation Society
JSP	Joint-Sacrificing Procedures
LCP	Locking Compression Plate
MBO	Modified Broström Operation
MDTA	Medial Distal Tibial Angle
OA	Osteoarthritis
ROM	Range of Motion
SMO	Supramalleolar Osteotomy
TLS	Tibial Lateral Surface
TTS	Tibiotalar Surface
VAS	Visual Analog Scale
